# Characteristics of Ionospheric Responses over China During the November 2023 Geomagnetic Storm and Evaluation of Positioning Performance of CORS in Low-Latitude Regions

**DOI:** 10.3390/s26072198

**Published:** 2026-04-02

**Authors:** Linghui Li, Youkun Wang, Junhua Zhang, Jun Tang, Fengjiao Yu, Jintao Wang, Zhichao Zhang

**Affiliations:** 1 Faculty of Land Resources Engineering, Kunming University of Science and Technology, Kunming 650093, China; lilinghui@stu.kust.edu.cn (L.L.); townjun@gmail.com (J.T.); wangjintao0102@stu.kust.edu.cn (J.W.); zhangzhichao@stu.kust.edu.cn (Z.Z.); 2 Kunming Surveying and Mapping Institute, Kunming 650051, China; shevchenko1123@163.com; 3 City College, Kunming University of Science and Technology, Kunming 650051, China; yfj_kust@kust.edu.cn

**Keywords:** geomagnetic storm, ionospheric, TEC, ionospheric response, RTK

## Abstract

This study used Global Navigation Satellite System (GNSS) observations from the China Crustal Movement Observation Network (CMONOC) and the Kunming Continuously Operating Reference Station (KMCORS) network to investigate ionospheric response characteristics over China during the geomagnetic storm of 4–6 November 2023, and to assess their impacts on CORS-based real-time kinematic (RTK) positioning performance in the low-latitude Kunming region. A quantitative assessment was conducted by integrating regional two-dimensional dTEC (%) maps over China, BeiDou Navigation Satellite System (BDS) Geostationary Earth Orbit (GEO) total electron content (TEC), the rate of TEC index (ROTI), and RTK positioning solutions to evaluate ionospheric disturbances, irregularity activity, and associated degradation in positioning performance. Results indicate that, during geomagnetic storms, ionospheric responses over China exhibit pronounced phase-dependent and latitudinal variations. During the second geomagnetic storm on 5–6 November, positive responses were dominant at mid-to-high latitudes, whereas alternating positive and negative responses were observed at low latitudes. During the recovery phase, the Kunming region successively experienced a positive ionospheric storm lasting approximately 10 h, followed by a negative ionospheric storm lasting about 7 h, with relative TEC variations reaching a maximum of approximately 90%. The GEO TEC time series was consistent with the temporal evolution of the two-dimensional dTEC (%), while ROTI increased markedly during the disturbance enhancement period (21:00 UT on 5 November to 07:00 UT on 6 November 2023). During periods of enhanced ionospheric response and irregularities, RTK positioning performance was observed to deteriorate markedly. The fixed-solution rate at medium-to-long baseline stations decreased from nearly 100% to close to 0%, accompanied by an increase in vertical positioning errors to approximately 20 cm, whereas short-baseline stations were only minimally affected. These results indicate that ionospheric disturbances during geomagnetic storms exert a pronounced impact on CORS-based RTK positioning services in the Kunming region, with the magnitude of this impact being closely related to baseline length.

## 1. Introduction

Geomagnetic storms result from enhanced solar wind–magnetosphere coupling that intensifies and disturbs magnetospheric current systems [1]. The ionosphere, a vital part of Earth’s upper atmosphere, spans altitudes from approximately 50 to 1000 km. Its existence results primarily from the ionization of atmospheric particles caused by solar radiation, particularly within the high-energy ultraviolet and X-ray spectral ranges [2]. The propagation of electromagnetic waves from the upper atmosphere to the Earth’s surface is influenced by ionospheric conditions [3,4,5]. In high-precision GNSS operations, ionospheric delay currently remains a major factor contributing to positioning and navigation inaccuracies. During geomagnetic storms, the injection of substantial energy and momentum into the upper atmosphere modifies the dynamics of the thermosphere and ionosphere. This subsequently triggers significant restructuring within the ionosphere, resulting in electron-density irregularities, variations in the equatorial ionospheric anomaly (EIA), and the development of travelling ionospheric disturbances (TIDs) [6]. These changes will weaken the GNSS signal, thus affecting the accuracy of satellite-based positioning and navigation [7,8].

The ionosphere response is affected by many factors, including the solar cycle, meteorological conditions, seasonal changes and local time [9,10,11,12,13]. The response of the ionosphere to geomagnetic storms may be completely different. According to whether the TEC or electron density decreases or increases during the geomagnetic storm, ionospheric storms can be classified as positive ionospheric storms or negative ionospheric storms [14,15]. In general, positive ionospheric storms are predominantly observed during the disturbance development phase and the early recovery phase and are characterized by increases in electron density or TEC. Negative ionospheric storms are characterized by decreases in electron density or TEC and are commonly observed during the post-disturbance phase in certain regions [16]. The response types, intensities, and durations may vary substantially across different events and regions.

Tanna and Pathak [17] investigated the impacts of geomagnetic storms on TEC and the ROTI at low latitudes, showing that ionospheric responses to the same storm may vary considerably across different locations (stations). Danilchuk et al. [18] analyzed the ROTI value and dynamic Precise Point Positioning (PPP) positioning error during the main phase of the extreme geomagnetic storm in May 2024. The results show that there is a strong positive correlation between the ionosphere index ROTI and the dynamic PPP error.

Over the past few decades, research on the impact of ionospheric responses during geomagnetic storms on GNSS positioning performance has primarily concentrated on PPP [19,20,21,22,23]. Luo et al. [24] examined the impact of moderate, strong, and super geomagnetic storms on PPP and reported that stations located in mid- to low-latitude regions exhibited comparatively smaller variations in positioning error. Lu et al. [20] evaluated the performance of multi-GNSS PPP during the St. Patrick’s Day geomagnetic storm and demonstrated that it performed significantly better than PPP using only GPS.

Compared with PPP, RTK requires stricter real-time performance and continuous availability in engineering applications, and its positioning performance is more susceptible to degradation under ionospheric disturbance conditions. Existing research on GNSS positioning performance during geomagnetic storms has primarily focused on PPP, whereas studies on RTK remain limited. The degradation of RTK positioning accuracy and availability directly affects the operational reliability and safety of practical applications, including surveying and mapping, engineering construction, and deformation monitoring. Previous studies have demonstrated that RTK positioning performance is significantly degraded during geomagnetic storms. Wielgosz et al. [25] observed that during the geomagnetic storm on 29 October 2003, the success rate of RTK instantaneous ambiguity resolution dropped to 31%, and on geomagnetic calm days, the success rate rose to 94%. Jacobsen and Schäfer [26] in their study of the network RTK positioning performance in Norway during the geomagnetic storm of 24 October 2011, showed that there was an exponential growth relationship between positioning error and the ROTI. Jacobsen and Andalsvik [27] further analyzed the impact of the St. Patrick’s Day geomagnetic storm from 17 to 18 March 2015 on the RTK positioning and ROTI of the same regional network. The results showed that as the ROTI value increased, the positioning error also increased rapidly. Follestad et al. [28] studied the performance of RTK from 2014 to 2016 and its relationship with the irregularity of the ionosphere, and revealed that there was a significant correlation between RTK positioning accuracy and ROTI, and the positioning error showed seasonal, daily and latitude changes. Zhang et al. [29] studied the RTK positioning performance at different levels of solar activity in the low latitudes of Hong Kong from 2013 to 2023. Their research results show that both RTK positioning rate and positioning accuracy are significantly correlated with solar activity. At the peak of solar activity, the positioning rate decreases significantly. The aforementioned studies indicate that RTK positioning performance in low-latitude regions is subject to varying degrees of degradation during geomagnetic storms; however, these issues warrant further systematic investigation.

Given the susceptibility of low-latitude regions to ionospheric anomalies, this study focuses on mid-to-low-latitude regions of China. It examines the severe geomagnetic storm affected by 3 interplanetary coronal mass ejections (ICMEs) that occurred from 4 to 6 November 2023 as a case study. This study examines ionospheric variations during geomagnetic storms and their impacts on positioning performance at both the regional and site scales. At the regional scale, GNSS observations from the CMONOC were used to characterize two-dimensional spatiotemporal variations in dTEC (%) over China. At the station scale, with a focus on the Kunming region, vertical TEC (VTEC) observations from BDS GEO satellite C03 were integrated with KMCORS RTK solutions to evaluate storm-time changes in positioning accuracy and ambiguity-fixing rate.

## 2. Data and Methods

### 2.1. Ionospheric Data

**Figure 1:**
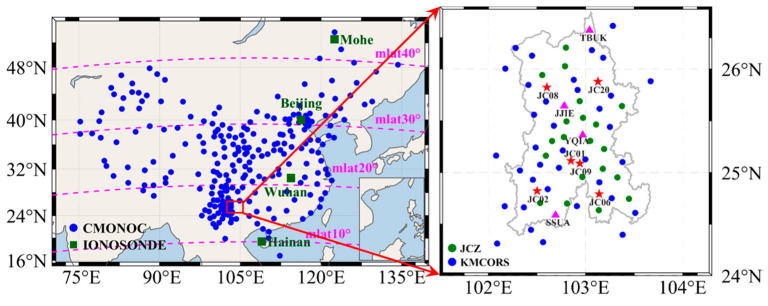
Distribution of GNSS and ionosonde stations. (**Left**) Blue dots represent the CMONOC stations, green squares denote ionosondes, and the red rectangle outlines the Kunming study area. (**Right**) Blue dots indicate the reference stations of the KMCORS network. Magenta triangles denote the four representative KMCORS reference stations (TBUK, JJIE, YQIA, and SSUA) selected for VTEC analysis based on the BDS GEO satellite C03. Green dots represent GNSS monitoring stations in the Kunming region (JCZ, consisting of 26 monitoring stations). Red stars mark the six monitoring stations (JC01, JC02, JC06, JC08, JC09, and JC20) used for RTK positioning performance evaluation in this study. Magenta dashed lines indicate magnetic latitude (mlat).

This study uses observational data from approximately 250 GNSS stations in the CMONOC and 48 stations in the KMCORS, with a sampling interval of 30 s for all stations. Figure 1 illustrates the spatial distribution of the GNSS stations and ionosondes: the left panel shows CMONOC stations as blue circles and ionosondes as solid green squares, whereas the right panel shows the KMCORS reference stations as blue circles and the Kunming monitoring stations as green circles. The four representative stations selected for VTEC analysis based on the BDS GEO satellite C03 are listed in Table 1.

This study uses dual-frequency GNSS observations to estimate ionospheric TEC. First, differential code bias (DCB) is corrected for in the dual-frequency geometry-independent pseudo-range combination to mitigate the effects of hardware delays on absolute TEC estimates. Subsequently, slant total electron content (STEC) is derived using a carrier-phase–smoothed pseudo-range method [30,31]. By introducing the Single-Layer Model (SLM), STEC is mapped to the zenith direction at the ionospheric pierce point (IPP) using a mapping function, thereby yielding the VTEC [32]. Assuming that free electrons are concentrated within a thin layer approximately 450 km above Earth’s surface, this study adopts an ionospheric shell height of H = 450 km [12,33]. For 4–6 November, the region over China was divided into a 1° × 1° latitude–longitude grid. With a temporal resolution of 1 min, the TEC values of all IPPs passing through each grid cell within each time window were collected. The arithmetic mean of these values was then assigned as the TEC value for that grid cell at that epoch, enabling the creation of two-dimensional TEC distribution maps without additional spatial interpolation. To obtain the overall relative trend of the ionospheric response during geomagnetic storms, this study selected 3 November as the quietest day based on data from the International Service for Geomagnetic Indices [34]. The grid TEC values of the corresponding times of the day is used as the background value, denoted as $TEC_{quiet}$.The differences dTEC between the grid TEC values on 4, 5, and 6 November and $TEC_{quiet}$ were then calculated. Finally, the relative change in grid TEC during the geomagnetic storm period, denoted dTEC%, was obtained by dividing dTEC by the background value $TEC_{quiet}$, as shown in Equation (1):

(1) $$dTEC(\%)=\frac{TEC-TEC_{\mathrm{quiet}}}{TEC_{\mathrm{quiet}}}\times 100\%$$

The ROTI was calculated using GNSS observation data from the Kunming regional monitoring stations to characterize ionospheric irregularity activity above the site during geomagnetic storm conditions. The ROTI was computed using a 5-min sliding time window with a sampling interval of 30 s, and the calculation formula is given as follows:

(2) $$ROT\left(i+1\right)=\frac{\Delta TEC_{t_{i}{,t}_{i+1}}}{t_{i+1}-t_{i}}=\frac{STEC_{i+1}^{j}-STEC_{i}^{j}}{t_{i+1}-t_{i}}$$

In this equation, ROT is derived from differences in STEC along the GNSS signal path. In $STEC_{i+1}^{j}$ and $STEC_{i}^{j}$, j denotes the satellite, while t denotes the epoch. $\Delta TEC_{t_{i}{,t}_{i+1}}$ represents the STEC difference between $STEC_{i+1}^{j}$ and $STEC_{i}^{j}$ over two consecutive epochs.

(3) $$\bar{ROT}=\frac{1}{N}\sum_{i=K-N+1}^{K}ROT\left(i\right)$$

where ROT denotes the TEC rate between adjacent epochs (TECU/min), while N is the number of epochs included in the statistic. With a 5-min time window and a 30-s sampling interval, N was set to 10.

(4) $$ROTI(k)=\sqrt{\frac{1}{N}\sum_{i=K-N+1}^{K}{\left(ROT\left(i\right)-\bar{ROT}\right)}^{2}}$$

In this study, a ROTI threshold of 0.5 TECU/min was adopted; values of ROTI=0.5 TECU/min were interpreted as indicating the presence of ionospheric irregularities [35].

### 2.2. Real-Time Kinematic

To evaluate the impact of ionospheric disturbances on RTK performance during strong geomagnetic storms, this study selects six representative monitoring stations (JC01, JC02, JC03, JC08, JC09, and JC20) for dual-constellation GPS/BDS RTK analysis. The observational data span 4–6 November 2023, with a sampling frequency of 1 Hz. All data were processed in the CGCS2000 reference frame. The locations of these monitoring stations are shown in Figure 1 as red pentagrams.

RTK positioning is performed using the open-source GNSS software the Real-Time Kinematic Library (RTKLIB, v2.4.2) [36], with Kunming Continuously Operating Reference Station (CORS) stations serving as the reference. In the processing, single-difference (SD) observations are first formed from measurements of the same epoch and satellite across receivers, and the SD observations from different satellites are then further differenced to generate double-difference (DD) measurements, thereby canceling receiver clock error terms. At the epoch level, DD pseud-orange and carrier-phase observations are jointly used for parameter estimation and integer ambiguity resolution to obtain fixed RTK solutions. The main processing parameters and strategies are summarized in Table 2.

The positioning error metric considered in this study includes two components: the horizontal error and the ellipsoidal height error (vertical error). The horizontal positioning error is defined as the discrepancy between the RTK-derived coordinates and the known reference coordinates in the horizontal plane and is computed as follows:

(5) $$Ds=\sqrt{{\left(dx\right)}^{2}+{\left(dy\right)}^{2}}$$

where dx and dy denote the differences between the known horizontal coordinate components and the RTK-derived coordinate components of the station in the east and north directions, respectively.

The ellipsoidal height error, denoted as Dh, is defined as the difference between the RTK-derived ellipsoidal height at a given epoch and the known reference ellipsoidal height.

The fixing rate is computed as the ratio of the number of epochs with successfully fixed ambiguities to the total number of theoretical epochs.

To quantitatively evaluate the overall positioning accuracy, the root-mean-square (RMS) values of the horizontal positioning root mean square error ($M_{s}$) and the ellipsoidal height root mean square error ($M_{h}$) over the observation period are computed as follows:

(6) $$\begin{array}{l} M_{s}=\sqrt{\left[\frac{DsDs}{n}\right]}\;\;\;\\ M_{h}=\sqrt{\left[\frac{DhDh}{n}\right]} \end{array}$$

where n denotes the number of epochs, $M_{s}$ represents the horizontal positioning root mean square error, and $M_{h}$ denotes the ellipsoidal height root mean square error.

## 3. Results and Analysis

### 3.1. Conditions of Solar and Geomagnetic Activity

**Figure 2:**
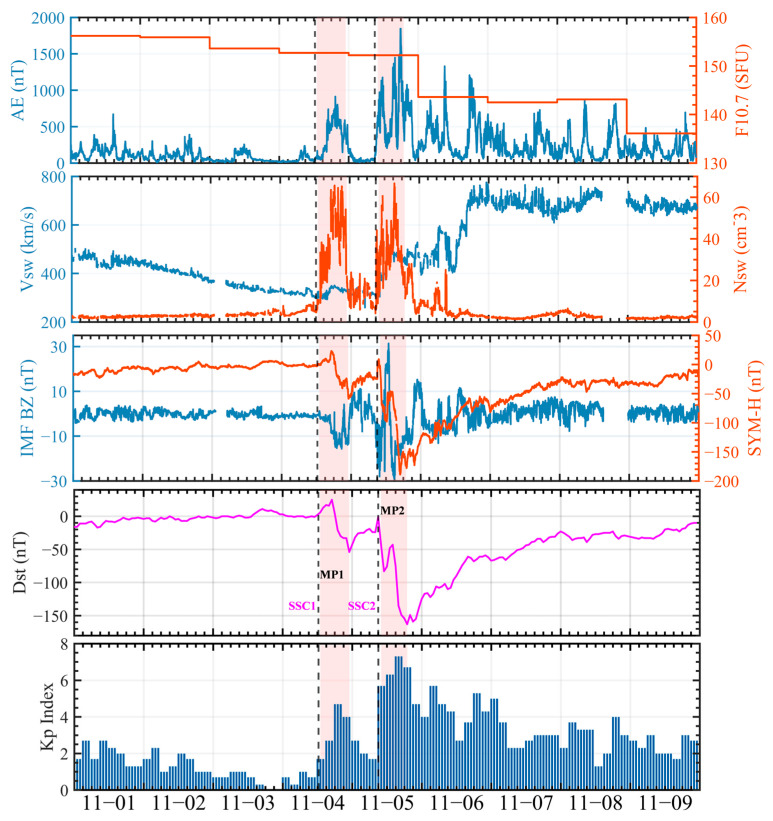
Variations in geomagnetic conditions from 1 to 9 November 2023. The gray dashed vertical lines mark the onset times of the two storm sudden commencements (SSC1 and SSC2), while the pink shaded regions highlight the corresponding main phases of the two geomagnetic storms and are labeled as MP1 and MP2, respectively.

The geomagnetic and solar activity index data used in this study were obtained from the NASA OMNIWeb database (https://omniweb.gsfc.nasa.gov/form/omni_min.html, accessed on 26 January 2026). To characterize the evolution of the near-Earth space environment during the geomagnetic storm from 1 to 9 November 2023, the time-series variations in the auroral electrojet index (AE), the solar radio flux index F10.7, solar wind speed (Vsw), solar wind proton density (Nsw), the Bz component of the interplanetary magnetic field (IMF), and the geomagnetic indices Dst, SYM-H, and Kp, were analyzed (see Figure 2).

Three days before the magnetic storm (1–3 November), the Solar and geomagnetic activity remained quiet, with the Dst index always above −20 nT and the Kp index always below 3 during this period. At about 13:00 UT on 4 November, the southward IMF Bz component reached the near-Earth environment. The speed of the solar wind gradually increased, and the proton density of the solar wind rose rapidly, marking the beginning of the main phase of the geomagnetic storm (13:00–23:00 UT on 4 November). During the main phase of geomagnetic storms, the F10.7 index stabilized at 152.7 SFU. At 18:00 UT on 4 November, the Kp index reached a peak of 4.7. At 18:40 UT, the solar wind speed reached a peak of 350.7 km/s. By 22:58 UT, the Dst index dropped to a minimum of −54 nT. During the recovery phase of the small geomagnetic storm, the IMF Bz component reversed to the north and reached a peak at 02:55 UT on 5 November, at about 9 nT.

After a small geomagnetic storm, the interstellar magnetic field’s Bz component moves south. At about 10:00 UTC on 5 November, the Bz component of the interplanetary magnetic field reached a minimum value of −25.5 nT. Subsequently, the Bz component of the interstellar magnetic field rotated to the north, reaching a maximum value of 31.38 nT. Then it moved south again, reaching the lowest point of −23.09 nT, and then turned north again, contributing to the occurrence of a double geomagnetic storm. During the main phase (10:00–19:00 UT on 5 November), the Bz component fluctuates between north and south. At 15:00 on 5 November, the Kp index reached a maximum of 7.3. At 11:00 UT, the Dst index dropped to −83 nT, and then at 14:00 UT Dst index rose to −43 nT, and the minimum reached −163 nT at 19:00 UT Dst index. The solar wind speed and proton density, as well as the Dst, SYM-H, and AE indices, exhibited consistent trends throughout this interval. Solar wind speed reached a maximum of 527 km/s, and the F10.7 index remained stable. In this geomagnetic storm event, the geomagnetism was at the level of large magnetic storm (Kp = 7.3) for 3 h and at the level of medium magnetic storm (Kp = 6) for 6 h. The geomagnetic storm lasted for two days. From 19:00 UT on 5 November, the IMF Bz component gradually turned northward, and the storm entered its recovery phase, with all indices progressively returning to quiet conditions. Before 03:00 UT on 7 November, the geomagnetic indices indicated that the geomagnetic field remained relatively active. Subsequently, the Kp index decreased to 3.7, the Dst index recovered to −69 nT, and the IMF Bz returned to a near-quiet level of approximately −1.1 nT, marking the conclusion of the geomagnetic storm. Based on the above characteristics, the second segment of the geomagnetic storm (following SSC2) was characterized by higher intensity and more pronounced disturbances. Accordingly, subsequent analyses in this study are primarily focused on the event of 5 November.

### 3.2. Ionospheric Response Characteristics

**Figure 3:**
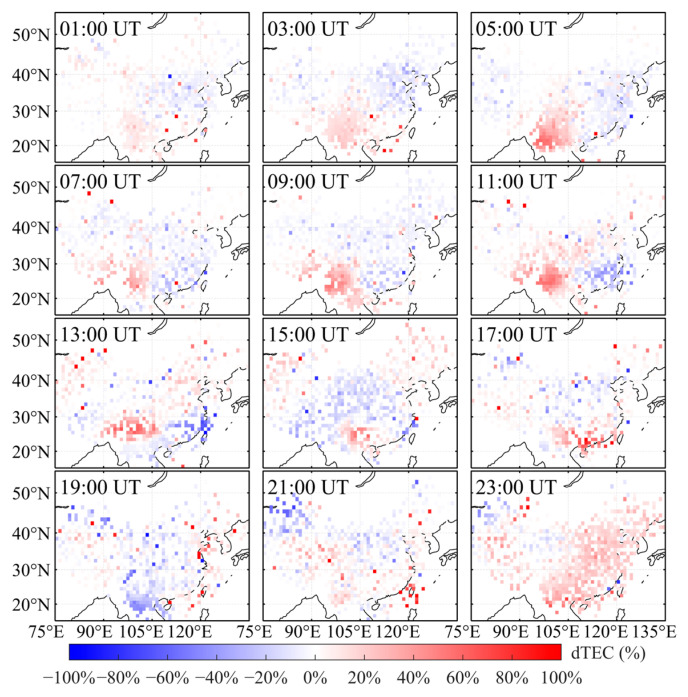
Two-dimensional map of dTEC (%) over China on 4 November 2023.

**Figure 4:**
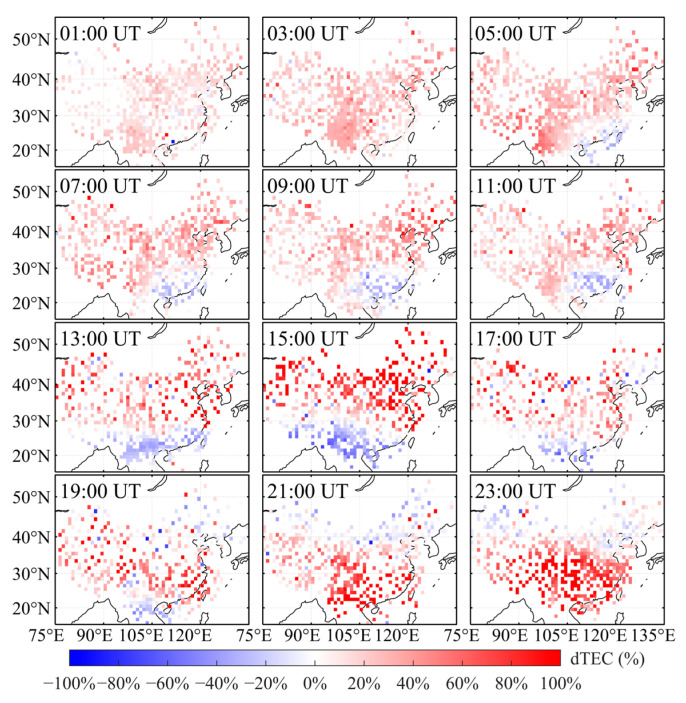
Two-dimensional map of dTEC (%) over China on 5 November 2023.

**Figure 5:**
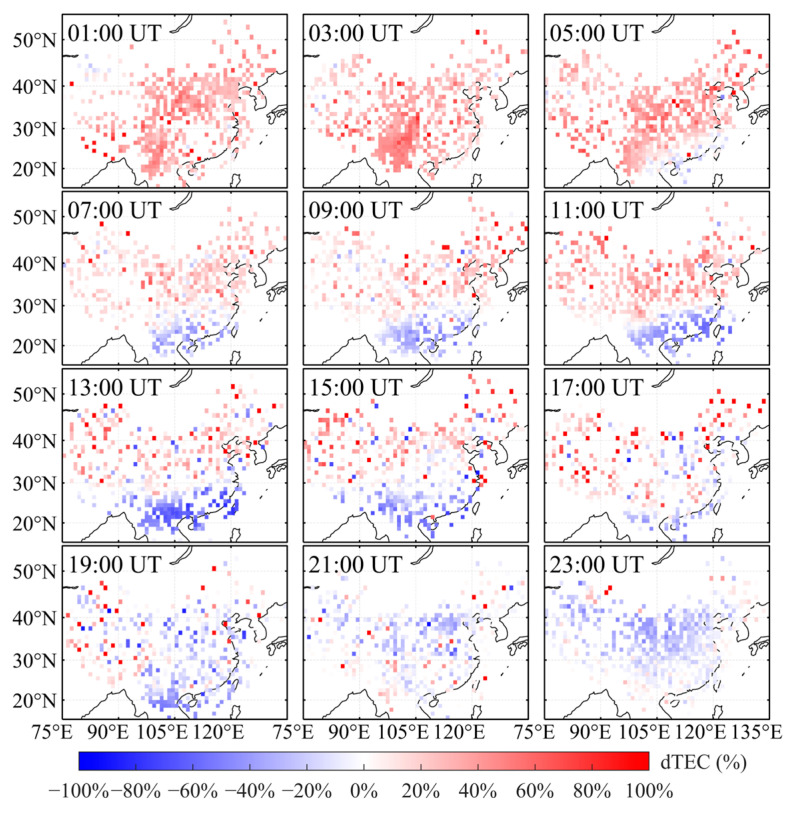
Two-dimensional map of dTEC (%) over China on 6 November 2023.

To analyze the ionospheric response over China during this geomagnetic storm, this study first derives TEC from the Chinese regional GNSS network. TEC on 3 November, a geomagnetically quiet day, is taken as the background, and TEC on 4–6 November (storm days) is evaluated relative to this reference. Using Equation (1), the relative TEC perturbation, dTEC (%), is then computed. On this basis, two-dimensional maps of dTEC (%) over China are generated for 4–6 November (Figure 3, Figure 4 and Figure 5) to investigate the spatiotemporal evolution of the ionospheric disturbance.

Figure 3 presents two-hourly maps of dTEC (%) over China on 4 November 2023. Overall, the ionosphere was in a weakly disturbed condition prior to the geomagnetic storm, with dTEC (%) values over most regions confined to approximately ±20%, and no persistent large-scale ionospheric storm was observed. At 01:00 UT, TEC began to increase over inland southwestern China, indicating the onset of a positive ionospheric storm. By 05:00 UT, the increase reached approximately 40%, after which it gradually diminished. Overall, the ionospheric disturbance during this interval exhibited a clear regional feature: the pronounced TEC enhancement was mainly confined to inland southwestern China, whereas at the national scale, most areas showed only moderate deviations from background levels. By 23:00 UT, TEC had returned to background values across most of the country.

Figure 4 presents 2-hourly maps of dTEC (%) over China on 5 November 2023. At 01:00 UT, the mid- and low-latitude regions were primarily characterized by weak positive disturbances, whereas the positive disturbance over southwestern China continued to intensify. Starting at 05:00 UT, a pronounced negative ionospheric storm developed over the southeastern coastal region and gradually extended westward along low latitudes. By 15:00 UT, this negative storm reached its maximum intensity, with dTEC (%) decreases of up to approximately −60%, forming a distinct low-latitude negative-storm belt. Meanwhile, sustained positive ionospheric disturbances persisted over mid-to-high latitudes (approximately 30–50° N). At 19:00 UT, TEC over low-to-mid latitudes (approximately 20–40° N) increased rapidly during the late phase of the geomagnetic storm, coinciding with the recovery of the geomagnetic indices. At 23:00 UT, the ionospheric storm reached its peak intensity, with a TEC amplification of approximately 95%. However, at this time, the ionospheric response over mid-to-high latitudes became negative. This indicates that the ionospheric response over the Chinese region exhibited alternating positive and negative phases.

Figure 5 presents two-hourly maps of dTEC (%) over China on 6 November 2023. These maps show that the positive ionospheric storm gradually weakened, while the low-latitude negative storm intensified and subsequently entered the recovery phase. From 01:00 to 05:00 UT, moderate positive disturbances persisted over mid-to-high latitudes (approximately 30–50° N). Activity south of 20°N remained relatively weak, with the ionosphere still transitioning from a positive to a negative storm phase. Starting at 07:00 UT, a negative storm emerged at low latitudes and extended westward along the low-latitude band. The negative disturbance intensified markedly and expanded laterally between 09:00 and 13:00 UT, with the maximum decrease in dTEC (%) reaching approximately −70%, while the positive disturbance at mid- and high latitudes gradually diminished. Thereafter, the intensity of the negative disturbance slowly decreased, and its spatial extent contracted. By 21:00–23:00 UT, TEC over most regions had returned to background levels, and the ionosphere had largely returned to near-quiet conditions.

**Figure 6:**
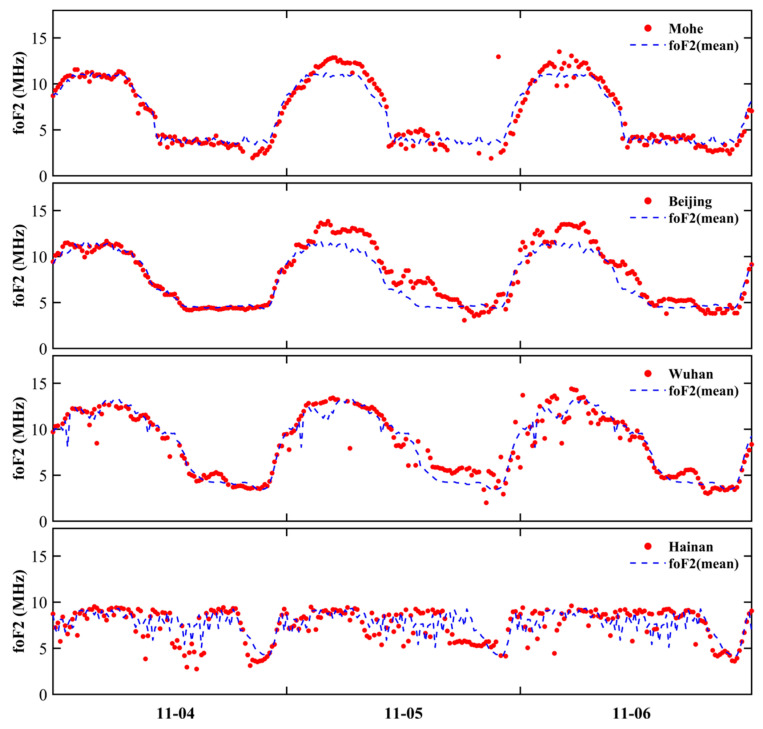
Variations in foF2 observed by ionosondes at Mohe (MH453), Beijing (BP440), Wuhan (WU430), and Hainan (HA419) from 4 to 6 November 2023.

The ionospheric response during geomagnetic storms is directly linked to increases in foF2, which therefore serves as an important parameter for assessing both the intensity and nature of the ionospheric reaction to geomagnetic disturbances [39]. To further verify the above GNSS TEC disturbance characteristics from the perspective of the F2-layer critical frequency, Figure 6 presents foF2 time series from four ionosonde stations—Mohe (MH453), Beijing (BP440), Wuhan (WU430), and Hainan (HA419)—for 4–6 November, together with the corresponding reference-day mean values. In the figure, red dots denote the foF2 observations on 4–6 November, whereas blue dashed lines indicate the mean foF2 values on the geomagnetically quiet days 1–3 November. The results show that, on 4 November, the observations agree well with the reference curves, and no obvious anomalies are evident in the foF2 data. On 5 November, foF2 at the four stations deviates markedly from the reference curves, and pronounced latitudinal differences become evident. During 03:00–17:00 UT, foF2 at all four stations is generally higher than the reference values, exhibiting a typical positive response with a clear latitudinal dependence: the enhancements at the high-latitude Mohe station and mid-latitude Beijing station are the most pronounced, with peak values elevated by about 1.5–3.0 MHz relative to the reference; the enhancement at Wuhan is smaller, whereas at Hainan the response is dominated by weak positive perturbations or values close to the background level, yielding a spatial pattern in which the intensity of the positive storm decreases with decreasing latitude. By 6 November, the ionospheric response undergoes a clear transition: the positive deviations at Mohe and Beijing weaken substantially overall and gradually return to near the reference levels, whereas Wuhan and Hainan exhibit persistent negative deviations during daytime, with foF2 at Hainan remaining continuously below the reference values and exhibiting a pronounced negative storm.

The above foF2 observation results are highly consistent with the space-time structure revealed by the two-dimensional dTEC (%) maps generated by GNSS (Figure 3, Figure 4 and Figure 5): In high latitudes, positive storms occur earlier and have a greater amplitude, while in low latitudes, the response is slightly delayed and negative storms last longer. This evolution of time from positive response to negative response, as well as the comparison between high-latitude and low-latitude areas, confirms the characteristic sequence of positive and negative ionosphere storms experienced in China during this geomagnetic event according to the critical frequency of the F2 layer, thus further confirming the reliability of GNSS-based ionosphere disturbance monitoring.

### 3.3. Impact on RTK Performance

**Figure 7:**
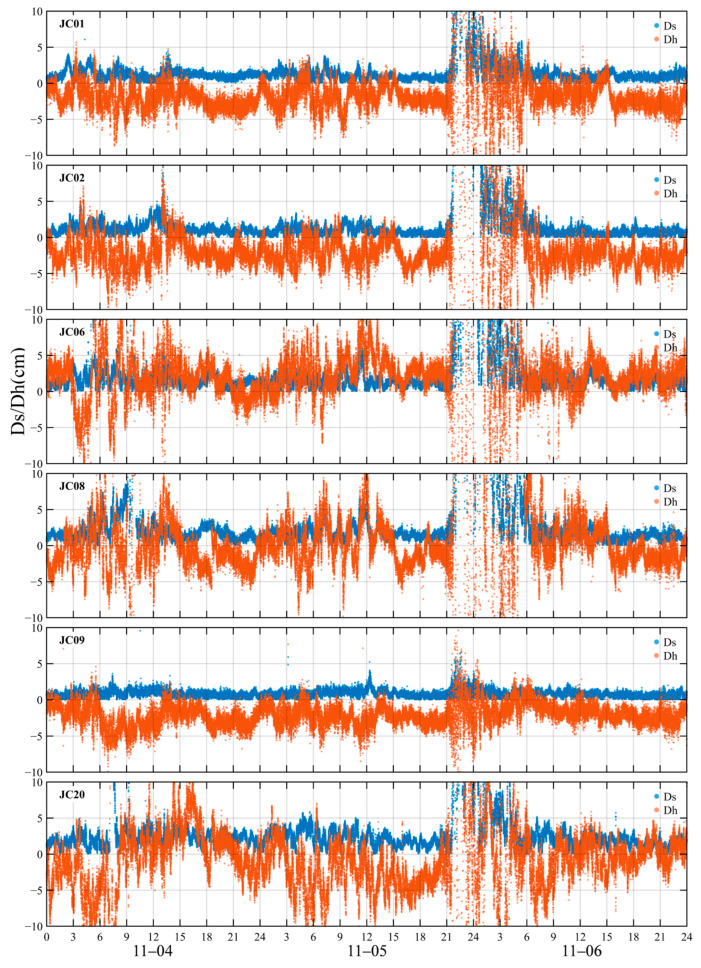
Scatter plots of RTK horizontal and ellipsoidal-height positioning error time series for six monitoring stations from 4 to 6 November 2023. Blue points represent the horizontal error (Ds), while orange points represent the ellipsoidal-height error (Dh). Positive and negative ellipsoidal-height errors indicate that the RTK-derived ellipsoidal height is higher or lower than the reference value, respectively.

This study used observational data from six monitoring stations within the Kunming CORS network, RTK horizontal and vertical positioning errors were calculated for each station, and RTK positioning performance in the Kunming region during geomagnetic storms was evaluated based on these error statistics. Given that RTK performance evaluation depends on the continuous availability of CORS network correction data and sufficient station density to support multi-station processing and comparative analyses, RTK positioning performance assessment was therefore confined to the coverage area of the Kunming CORS network. Ionospheric response analysis was conducted to characterize spatiotemporal variations at the regional scale over China, thereby providing a contextual reference for interpreting regional RTK performance variations. To evaluate the impact of ionospheric responses during geomagnetic storm events on RTK positioning accuracy, a statistical analysis was conducted using RTK positioning results from CORS monitoring stations in the Kunming region. As shown in Figure 7, RTK positioning errors fluctuated markedly between 03:00 and 13:00 UT on 4 November, with vertical errors generally exhibiting larger variations than horizontal errors. Monitoring stations JC01 and JC09 showed the least fluctuation, especially monitoring station JC09, whose horizontal error remained almost unchanged and within 2.0 cm, and whose vertical error remained within 5.0 cm; monitoring station JC20 showed the greatest fluctuation in RTK positioning error, with the vertical error reaching a maximum of 15 cm. The vertical error between monitoring stations JC08 and JC06 is about 10 cm, while the horizontal error of JC08 reached 8 cm. After 13:00 UT, the horizontal and vertical positioning errors of RTK gradually decreased, with the horizontal and vertical errors being better than 2.0 cm and 5.0 cm, respectively. Starting at 21:00 UT on 5 November, the RTK horizontal and vertical positioning errors of the monitoring stations increased sharply. Except for monitoring station JC09, the horizontal and vertical positioning errors of the other monitoring stations increased sharply by about 10 cm and 20 cm, respectively. The positioning error of monitoring station JC09 increased slightly but within 5 cm. Before 21:00 UT, the positioning error of the monitoring station fluctuated slightly, with the error of monitoring station JC20 being relatively larger. On 6 November, at 07:00, the positioning error of the UT monitoring station gradually decreased, and its horizontal and vertical errors recovered to 3.0 cm and 5.0 cm, respectively. It is worth noting that the RTK positioning error of monitoring station JC09 fluctuated the least throughout the entire geomagnetic storm, remaining stable.

## 4. Discussion

This study primarily focuses on the observed characteristics of ionospheric disturbances and their impacts on RTK positioning performance. Due to the lack of direct observational data on electric fields, neutral winds, and thermospheric composition, the specific physical driving mechanisms of ionospheric disturbances are not investigated in this study. Based on observational data from the CMONOC and KMCORS networks, a two-dimensional dTEC (%) map of China was constructed to analyze ionospheric response characteristics during the geomagnetic storm period from 4–6 November 2023. The results indicate that, as shown in Figure 2, two consecutive geomagnetic storm events occurred between 4 and 6 November 2023. The event on 4 November was a relatively weak geomagnetic storm, whereas the event on 5 November was of moderate intensity. Figure 3, Figure 4 and Figure 5 show that only weak ionospheric disturbances occurred over China on 4 November, with a predominantly positive response lasting approximately 10 h. In contrast, pronounced ionospheric anomalies were observed over China on 5–6 November. The second main phase of the geomagnetic storm on 5 November (following SSC2) was characterized by pronounced spatial inhomogeneity, with alternating distributions of positive and negative responses. Positive responses dominated at mid-to-high latitudes over China, and this main phase persisted for approximately 9 h. During the early recovery phase of the second geomagnetic storm, the overall ionospheric response at mid-to-low latitudes over China remained predominantly positive. Subsequently, alternating weak ionospheric disturbances of positive and negative polarity continued to be observed over China, with negative responses gradually becoming dominant.

**Figure 8:**
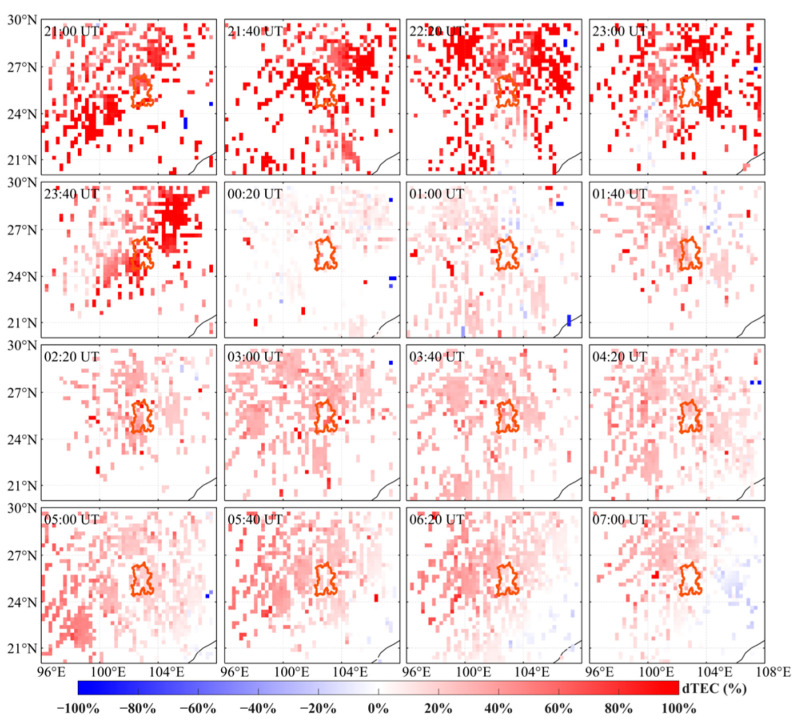
Two-dimensional distributions of dTEC (%) over Kunming and the surrounding region from 21:00 to 07:00 UT on 5–6 November 2023. The orange line delineates the administrative boundary of Kunming.

**Figure 9:**
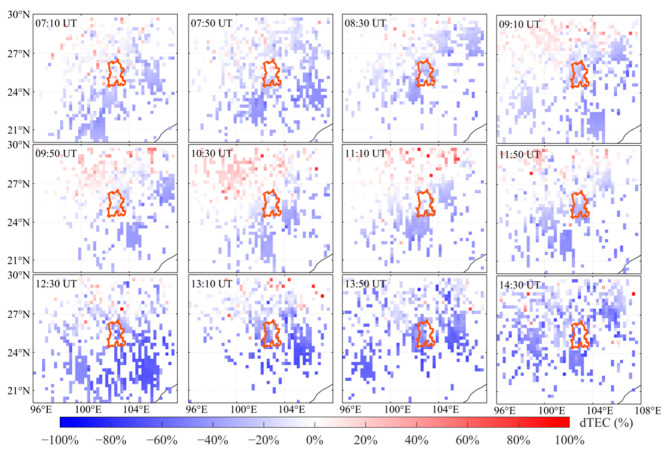
Two-dimensional distributions of dTEC (%) over Kunming and the surrounding region from 07:10 to 14:30 UT on 6 November 2023. The orange line delineates the administrative boundary of Kunming.

To further analyze the evolution of positive and negative storms over the Kunming region during the recovery phase, two-dimensional dTEC (%) maps were generated at 40 min intervals. Figure 8 shows two-dimensional dTEC (%) distributions for Kunming and the surrounding region from 21:00 to 07:00 UT on 5–6 November 2023. During the recovery phase of the geomagnetic storm, a distinct positive ionospheric disturbance emerged in the northern and western regions of Kunming, with the disturbance intensity continuing to increase steadily. Between 21:00 and 23:40 UT, the positive disturbance over Kunming continued to intensify, with the amplitude of the positive dTEC anomaly increasing by approximately 90% relative to the reference level. On 6 November, as the disturbance propagated, the intensity of the positive-phase disturbance over Kunming began to weaken and eventually dissipated. Subsequently, the Kunming region entered a pronounced negative storm phase. As can be seen from Figure 9, a clear negative phase disturbance was observed over Kunming from 07:10 to 14:30 UT. A clear negative disturbance has been present over Kunming, with the disturbance occurring earlier in the low-latitude region than in the high-latitude region. From 12:30 to 13:50 UT, the negative phase disturbance gradually intensified, and after 13:50 UT, its intensity gradually decreased as it propagated. By 14:30 UT, the negative disturbance had gradually weakened. Overall, the Kunming region first experienced a positive storm that intensified and then weakened, and subsequently transitioned into a pronounced negative storm phase lasting about 9 h during the daytime of 6 November.

**Figure 10:**
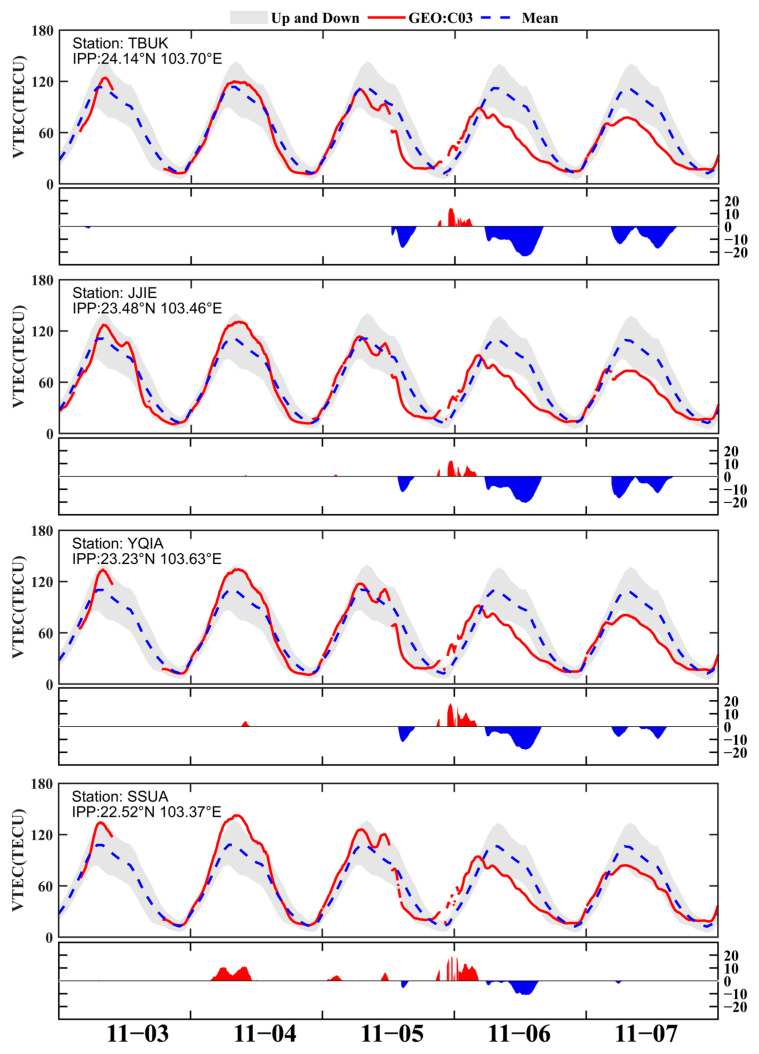
Temporal variations in VTEC derived from BDS GEO C03 satellite observations over the Kunming region from 4 to 6 November 2023 at four CORS stations.

To further investigate ionospheric response characteristics at the site scale in the Kunming region, the interquartile range (IQR) method was employed to detect anomalies in the GEO VTEC time series. Because the BDS GEO satellite C03 provides excellent coverage over the Kunming region at a high elevation angle, more continuous and stable VTEC measurements can be obtained; therefore, data from GEO C03 were used in the analysis. Figure 10 presents GEO C03 VTEC observations from four KMCORS reference stations (ordered from north to south according to the IPP latitude: TBUK, JJIE, YQIA, and SSUA) from 3 to 7 November, together with the corresponding background reference curve. The solid red line represents the VTEC observed by GEO C03, the dashed blue line indicates the Global Ionospheric Map (GIM) moving mean computed from the 30 days preceding the observation date, and the gray shaded area denotes the upper and lower anomaly boundaries defined by the IQR criterion within the moving window. The red and blue shaded regions in ∆TEC correspond to the positive and negative storm-time responses, respectively.

As shown in Figure 10, during the quiet period from 3–4 November 2023, the observed TEC did not display any evident disturbance features. After the geomagnetic storm entered its recovery phase, the GEO-derived VTEC increased sharply. Between 21:00 UT on 5 November 2023 and 07:00 UT on 6 November 2023, the TEC reached its peak values across the monitoring stations. The red regions in the figure indicate periods of positive ionospheric response during this interval. The largest ∆TEC enhancement was 19.2 TECU at the SSUA station, whereas the smallest enhancement was 14.3 TECU at the TBUK station. The positive responses at SSUA and YQIA persisted for approximately 10 h, while those at JJIE and TBUK were notably shorter. In addition to positive responses, negative ionospheric disturbances were observed over Kunming from 07:00 to 14:30 UT on 6 November. These disturbances were more pronounced at the TBUK and JJIE stations, but weaker at YQIA and SSUA. Compared with the positive phase, the minimum ∆TEC depletion was −11 TECU at SSUA, whereas the largest depletion reached −23.4 TECU at TBUK. The negative disturbances at SSUA and YQIA lasted for a shorter interval, while those at JJIE and TBUK were stronger and persisted for nearly 7 hours. This comparison shows that the GNSS-derived two-dimensional dTEC (%) maps are consistent with the disturbance characteristics reflected in the GEO- derived VTEC time series.

**Figure 11:**
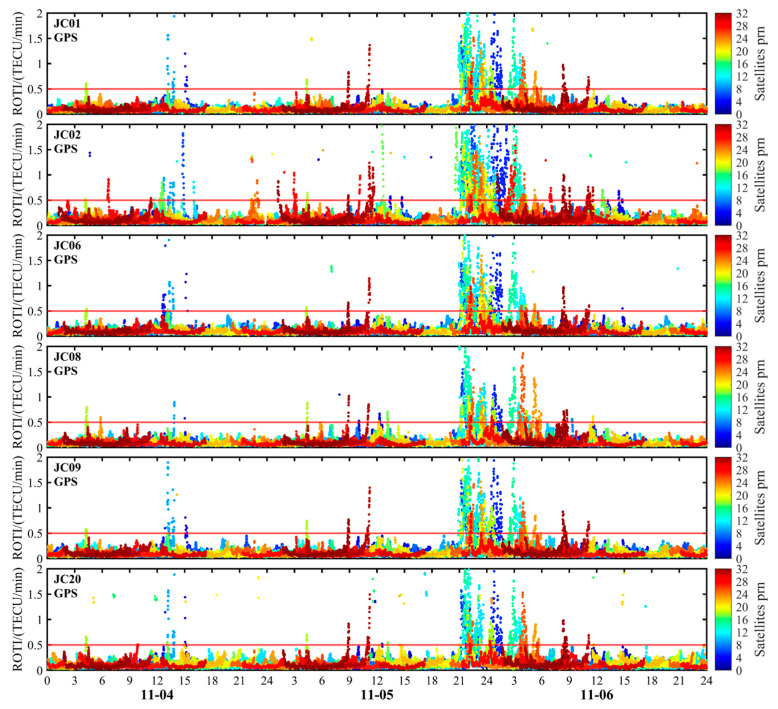
Time-series variation in ROTI derived from GPS observations at the Kunming monitoring station during 4–6 November 2023. The red horizontal line indicates the threshold value.

**Figure 12:**
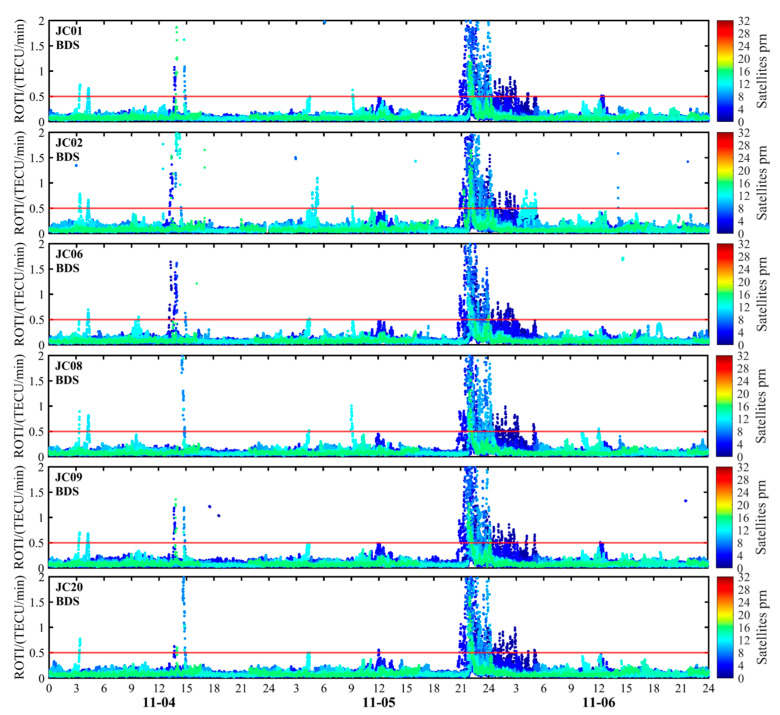
Temporal variation in the BDS ROTI sequence observed at the Kunming monitoring station during 4–6 November 2023, illustrating ionospheric irregularity activity associated with the geomagnetic storm. The red horizontal line indicates the threshold value.

This study employs GEO VTEC and dTEC% to characterize large-scale background variations in the Kunming region’s ionosphere during geomagnetic storms at the regional scale. Furthermore, it utilizes the ROTI index from monitoring stations to analyze small-scale ionospheric irregularities. Figure 11 and Figure 12 display the ROTI time series obtained from observations of the GPS and BDS systems at six monitoring stations in the Kunming region. Prior to the onset of the main phase of the first geomagnetic storm, before 13:00 UT on 4 November, the ionosphere above the six monitoring stations remained generally calm. The ROTI values mostly stayed at low levels with minimal fluctuations, indicating a relatively stable ionospheric condition. Starting around 13:00 UT, ROTI values for some satellites increased significantly, indicating heightened short-term ionospheric disturbance activity. During the second geomagnetic storm on 5 November, ionospheric activity significantly intensified at all monitoring stations during the early recovery phase. The ROTI value rose continuously from 21:00 UT to 07:00 UT the following day, with ionospheric scintillation activity persisting for nearly 10 h—particularly between 21:00 and 23:40 UT. The ROTI sequence exhibited pronounced fluctuations, with ROTI values from certain satellites increasing significantly, indicating distinct ionospheric scintillation phenomena. This feature was clearly evident at all six monitoring stations, indicating a significant increase in ionospheric instability at the station scale during the magnetic storm period in the Kunming region.

**Figure 13:**
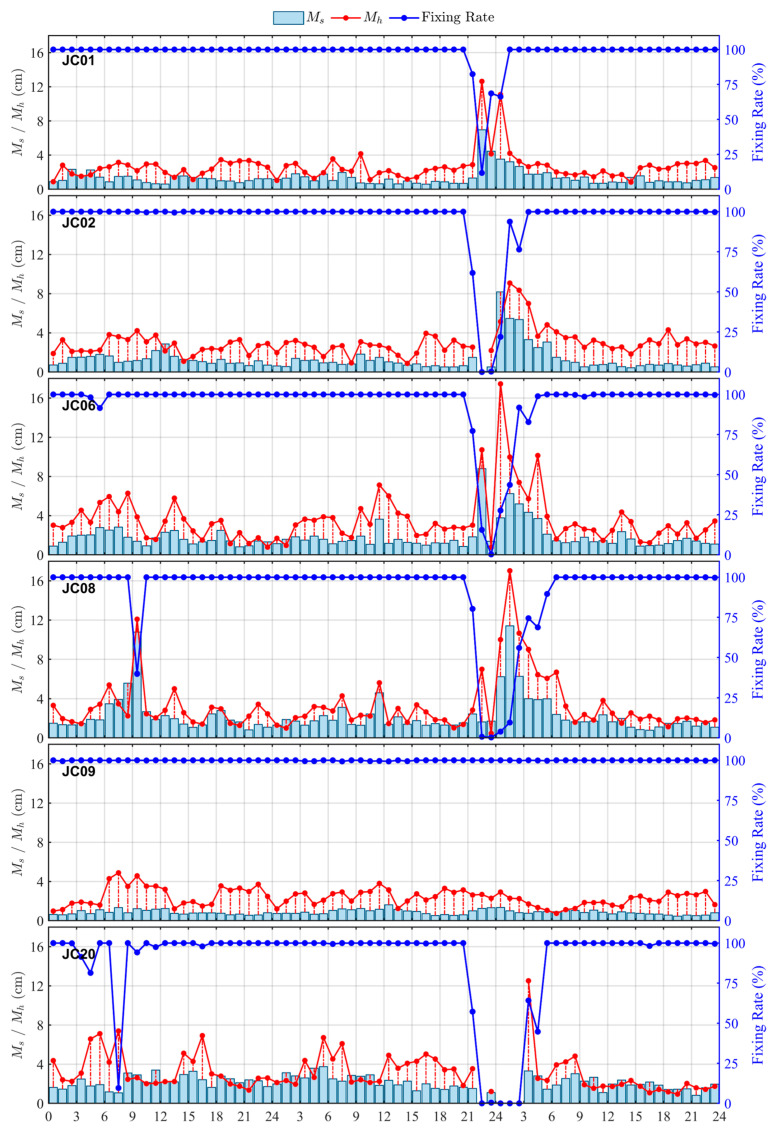
Temporal variations in RTK positioning accuracy and fixing rate at the monitoring stations from 4 to 6 November 2023.

To further analyze the temporal characteristics of RTK positioning accuracy, this study first applied a ±0.15 m clipping threshold to the X, Y, and H components of each monitoring station in the KMCORS network and removed gross errors. Subsequently, it calculated the 95% confidence-level horizontal RMS error ($M_{s}$), the vertical RMS error ($M_{h}$), and the fixing rate on an hourly basis, and plotted the corresponding 72 h time-series curves (see Figure 13). The results show that the short-baseline monitoring station JC09 exhibited the most stable performance throughout the geomagnetic storm, with $M_{s}$ consistently below 1.0 cm and $M_{h}$ below 3.0 cm, clearly outperforming the other monitoring stations. During the minor geomagnetic storm on 4 November, the fixing rate at most monitoring stations remained close to 100%, with only slight fluctuations in $M_{s}$ and $M_{h}$. At only a few monitoring stations did the fixing rate drop sharply before the main interval (03:00–10:00 UT), with monitoring stations JC08 and JC20 experiencing significant decreases to 40% and 10%, respectively. During the main phase (13:00–23:00 UT), the fixing rate exhibited noticeable variations, whereas $M_{s}$ and $M_{h}$ remained stable. During the main phase of the second geomagnetic storm on 5 November (10:00–19:00 UT), the fixing rate at all monitoring stations remained stable. During the early recovery phase, beginning at 21:00 UT, as ionospheric disturbances intensified, the fixing rate at all monitoring stations—except for the short-baseline monitoring station JC09—dropped sharply from nearly 100% to almost 0% within a few hours. Starting at 00:00 UT on 6 November, the fixing rates at monitoring stations JC01, JC02, JC06, and JC08 began to recover gradually. Monitoring station JC01 was the first to recover to 100% at 02:00 UT, followed by monitoring station JC02 and JC06 in turn. By 07:00 UT, the fixing rate at all monitoring stations had returned to normal levels. It is worth noting that the fixing rate of monitoring station JC20 remained at 0% throughout this period, its ambiguity could not be fixed, the positioning performance collapsed, and the RTK solution could only output floating-point solutions or single-point solutions, thus failing to obtain high-precision fixed solutions. Meanwhile, the vertical and horizontal errors of all monitoring stations increased significantly, with peak values reaching approximately 12 cm and 15 cm, respectively. It is worth noting that the increase and fluctuation of the vertical error were much greater than those of the horizontal error. The sharp decrease in the fixing rate and the significant increase in the error, was highly synchronized in time, with both reaching their extreme values during the same period. By 07:00 UT on 6 November, the fixing rate of each station gradually recovered to 100%, and both $M_{s}$ and $M_{h}$ had returned to near-normal levels.

Differences in baseline lengths between monitoring stations further underscore the influence of ionospheric delay errors on RTK positioning performance. As shown in Table 3, the short baseline measuring monitoring station JC09 is highly consistent with the spatial environment of the reference station, and the received RTK correction values are closer to the true spatial error state. Its ambiguity-fixing rate is always close to 100%, and the horizontal and vertical errors, represented by $M_{s}$ and $M_{h}$ remain stable within 1 cm and 3 cm, respectively. In contrast, as baseline length increases, spatial non-uniformity in the ionosphere becomes progressively more pronounced. Residual ionospheric delay errors in double-difference observations become increasingly difficult to fully mitigate, thereby reducing the spatial representativeness of the correction values. During geomagnetic storms, particularly during periods of enhanced ionospheric activity, these stations are more likely to experience degraded ambiguity resolution and a higher proportion of float solutions. Consequently, positioning accuracy and solution stability are substantially degraded.

During the first geomagnetic storm on 4 November, although some ionospheric disturbance was present, the overall disturbance intensity remained limited. The ambiguity-fixing rate at all monitoring stations remained close to 100%. RTK positioning performance remained generally stable, with only minor fluctuations in positioning errors. During this phase, ROTI remained consistently low, indicating weak station-scale ionospheric inhomogeneity.

In contrast, during the second geomagnetic storm on 5 November, ionospheric disturbances exerted a more pronounced impact on RTK positioning performance. During the initial recovery phase (21:00–23:40 UT), the Kunming region exhibited a pronounced positive increase in dTEC% (peaking at approximately 90%; Figure 8), accompanied by a marked enhancement in regional-scale ionospheric delay. Meanwhile, ROTI intensified simultaneously across multiple monitoring stations, indicating a marked increase in station-scale short-term ionospheric irregularities and a substantial enhancement in ionospheric spatial inhomogeneity. At this stage, residual ionospheric delay errors at long-baseline stations were substantially amplified. RTK corrections were unable to accurately represent the true ionospheric conditions at the monitoring station locations, leading to increased instability in coordinate estimation. This resulted in a marked decline in fixed-solution rates, with positioning solutions exhibiting a higher proportion of float solutions and increased coordinate deviations.

Subsequently, between 23:40 and 07:00 UT on the following day, as the geomagnetic storm gradually weakened, ROTI levels steadily declined. TEC levels and ionospheric spatial gradients decreased, leading to a gradual reduction in ionospheric errors. Consequently, the RTK ambiguity-fixing rate and positioning accuracy exhibited a gradual recovery. Upon entering the negative TEC phase on 6 November, dTEC% indicated a substantial reduction in regional-scale TEC (approximately 70%). ROTI did not exhibit sustained enhancement, and the RTK fixing rate did not experience a substantial decline. Positioning performance remained generally stable, with the maximum horizontal error reaching approximately 12 cm. At this stage, ionospheric spatial inhomogeneities were relatively weak, resulting in minimal differences in ionospheric delay between reference and monitoring stations. Consequently, the spatial correlation of RTK corrections was well preserved.

It should be noted that the period of significantly enhanced ROTI closely overlaps with the interval of reduced RTK ambiguity fixed-solution rates and increased positioning errors, and this concurrence occurs primarily during the active geomagnetic storm over the Kunming region. This indicates that, although regional-scale GEO VTEC and dTEC% capture large-scale ionospheric responses, station-level small-scale disturbances become more influential for high-precision RTK positioning during geomagnetic storms. Additionally, GPS- and BDS-derived ROTI exhibited synchronous enhancements during key periods, providing consistent cross-system evidence of ionospheric irregularity activity. Although the two systems differed in peak amplitude and duration, their primary enhancement periods were well aligned, indicating that the observed disturbance was unlikely to have been caused by anomalies in a single system or by individual satellites.

## 5. Conclusions

This study systematically examines the geomagnetic storm event that occurred from 4 to 6 November 2023, with a focus on three key aspects: regional ionospheric response characteristics over China, ionospheric responses at the Kunming site (including GEO VTEC and ROTI), and variations in RTK positioning performance. The main conclusions are summarized as follows:

1. During geomagnetic storms, ionospheric responses over China exhibit pronounced phase-dependent and zonal variations. On 4 November, disturbances were relatively weak, with positive responses predominating. From 5 to 6 November, disturbances intensified substantially, with alternating positive and negative responses observed across regions, although positive responses remained dominant at mid-to-high latitudes. Temporal variations in GEO VTEC at the Kunming stations were generally consistent with the regional dTEC (%) evolution, reflecting the phase-dependent changes in ionospheric responses over the region.
2. ROTI increased markedly during periods of enhanced disturbances, indicating elevated ionospheric irregularity activity above the monitoring stations. The increase in ROTI was temporally associated with a clear degradation in RTK solution quality, primarily manifested as reduced fixing rates, a higher proportion of float and single-point solutions, and abrupt increases in positioning errors. These results suggest that enhanced ionospheric irregularities substantially degrade RTK solution stability.
3. RTK positioning performance was substantially degraded during periods of enhanced ionospheric responses and irregularity activity, and the severity of degradation was closely related to baseline length. During the recovery phase of the second geomagnetic storm, medium-to-long baseline monitoring stations exhibited pronounced performance degradation: ambiguity fixing rates decreased from nearly 100% to close to 0%, while the mean vertical error increased to approximately 20 cm. The results indicate that longer baselines are associated with larger double-difference ionospheric residuals between reference and monitoring stations, which can more strongly perturb ambiguity estimation and coordinate solutions, thereby degrading RTK positioning performance.

## Figures and Tables

**Table 1:** Geographic and Geomagnetic Coordinates of GNSS Stations Equipped with BDS GEO Receivers in the Kunming Region.

Reference Station	Geographic Coordinates	Geomagnetic Coordinates
TBUK	26.37° N, 103.04° E	17.17° N, 175.95° E
JJIE	25.64° N, 102.78° E	16.44° N, 175.70° E
YQIA	25.36° N, 102.97° E	16.16° N, 175.87° E
SSUA	24.59° N, 102.69° E	15.39° N, 175.60° E

**Table 2:** Main configuration parameters for RTK processing.

Item	Parameter/Method
Processing strategy	DD
Sampling rate	1 Hz
Constellations and frequency bands	Dual-frequency GPS/BDS
Ephemeris and clock products	Broadcast ephemerides and satellite clock corrections (decoded from RTCM messages)
Ionospheric correction	Ionospheric error mitigation for short baselines based on the double-difference model
Tropospheric model	Saastamoinen model [37] with Global mapping function [38]
Ambiguity resolution	Integer-fixed solution (ratio threshold = 3)
Coordinate reference frame	CGCS2000

**Table 3:** Monitoring stations, reference stations, and baseline lengths.

Monitoring Station	Reference Station	Baseline Length (km)
JC09	CSYX	0.96
JC01	CSFX	4.17
JC02	BIJI	11.14
JC06	YLIA	12.56
JC08	YNCH	15.28
JC20	GONS	20.18

Note: Baseline length denotes the horizontal distance from the reference station to the monitoring station; short baselines are <5 km, medium baselines are 10–15 km, and long baselines are >15 km.

## Data Availability

The GNSS data are from CMONOC and the Kunming CORS, the foF2 data are from the Meridian Project (https://dcstatus.meridianproject.ac.cn/, accessed on 26 January 2026), and the geomagnetic and solar activity index data are from NASA (https://omniweb.gsfc.nasa.gov/form/omni_min.html, accessed on 26 January 2026).

## References

[1] Gonzalez W.D., Joselyn J.A., Kamide Y., Kroehl H.W., Rostoker G., Tsurutani B.T., Vasyliunas V.M. (1994). What is a geomagnetic storm?. J. Geophys. Res. Space Phys..

[2] Aa E., Zhang S.-R., Erickson P.J., Wang W., Qian L., Cai X., Coster A.J., Goncharenko L.P. (2023). Significant Mid- and Low-Latitude Ionospheric Disturbances Characterized by Dynamic EIA, EPBs, and SED Variations During the 13–14 March 2022 Geomagnetic Storm. J. Geophys. Res. Space Phys..

[3] Koucká Knížová P., Laštovička J., Kouba D., Mošna Z., Podolsk K., Podolská K., Šindelářová T., Chum J., Rusz J. (2021). Ionosphere influenced from lower-lying atmospheric regions. Front. Astron. Space Sci..

[4] Klobuchar J.A. (1987). Ionospheric time-delay algorithm for single-frequency GPS users. IEEE Trans. Aerosp. Electron. Syst..

[5] Yasyukevich Y., Astafyeva E., Padokhin A., Podlesnyi A. (2018). The 6 September 2017 X-class solar flares and their impacts on the ionosphere, GNSS, and HF radio wave propagation. Space Weather.

[6] Tsurutani B.T., Verkhoglyadova O., Mannucci A., Saito A., Araki T., Yumoto K., Tsuda T., Abdu M.A., Sobral J.H.A., Gonzalez W.D. (2008). Prompt penetration electric fields (PPEFs) and their ionospheric effects during the great magnetic storm of 30–31 October 2003. J. Geophys. Res. Space Phys..

[7] Zhang S., He L., Wu L. (2020). Statistical study of loss of GPS signals caused by severe and great geomagnetic storms. J. Geophys. Res. Space Phys..

[8] Luo X., Du J., Lou Y., Gu S., Yue X., Liu J., Chen B. (2022). A method to mitigate the effects of strong geomagnetic storm on GNSS precise point positioning. Space Weather.

[9] Liu L., Zou S., Yao Y., Aa E. (2020). Multi-scale ionosphere responses to the May 2017 magnetic storm over the Asian sector. GPS Solut..

[10] Yao Y., Liu L., Kong J., Zhai C. (2016). Analysis of the global ionospheric disturbances of the March 2015 great storm. J. Geophys. Res. Space Phys..

[11] Tang J., Yang D., Liu H. (2024). Study of Chinese regional ionospheric TEC response to magnetic storms during April 23–25, 2023. GPS Solut..

[12] Wang J., Tang J., Xu C., Zhang L., Wang Y. (2025). Ionospheric Disturbances Detection Based on BDS-GEO Observations With Prophet Model Over China: A Case Study of May 2017 Geomagnetic Storm. J. Geophys. Res. Space Phys..

[13] Wang Y., Yao Y., Kong J., Shan L. (2023). Ionospheric Disturbances Triggered by China’s Long March 2D Rocket. IEEE J. Sel. Top. Appl. Earth Obs. Remote Sens..

[14] Lei J., Huang F., Chen X., Zhong J., Ren D., Wang W., Yue X., Luan X., Jia M., Dou X. (2018). Was magnetic storm the only driver of the long-duration enhancements of daytime total electron content in the Asian-Australian sector between 7 and 12 September 2017?. J. Geophys. Res. Space Phys..

[15] Matamba T.M., Habarulema J.B., McKinnell L.A. (2015). Statistical analysis of the ionospheric response during geomagnetic storm conditions over South Africa using ionosonde and GPS data. Space Weather.

[16] Fuller-Rowell T., Codrescu M., Moffett R., Quegan S. (1994). Response of the thermosphere and ionosphere to geomagnetic storms. J. Geophys. Res. Space Phys..

[17] Tanna H.J., Pathak K.N. (2014). Longitude dependent response of the GPS derived ionospheric ROTI to geomagnetic storms. Astrophys. Space Sci..

[18] Danilchuk E., Yasyukevich Y., Vesnin A., Klyusilov A., Zhang B. (2025). Impact of the May 2024 Extreme Geomagnetic Storm on the Ionosphere and GNSS Positioning. Remote Sens..

[19] Bezerra L.d.S., de Oliveira P.S., Krueger C.P. (2025). Performance analysis of multi-GNSS PPP under the effects of extreme geomagnetic event: A case study of Mother’s Day solar storm (10–15 May 2024). Adv. Space Res..

[20] Lu Y., Wang Z., Ji S., Chen W. (2020). Assessing the positioning performance under the effects of strong ionospheric anomalies with multi-GNSS in Hong Kong. Radio Sci..

[21] Geng W., Huang W., Liu G., Liu S., Luo B. (2022). Assessing the Kinematic GPS Positioning Performance Under the Effect of Strong Ionospheric Disturbance Over China and Adjacent Areas During the Magnetic Storm. Radio Sci..

[22] Li W., Song S., Cheng N. (2022). Observation of the Impacts of Solar Flares on the Ionosphere and Precise point Positioning in South America Around the Halloween 2021. Proceedings of the 2022 IEEE International Geoscience and Remote Sensing Symposium (IGARSS 2022).

[23] Zhou W., Yuan Y., Tang C., Meng Y., Chen Y. (2024). Ionosphere disturbances on GNSS signal and positioning performance: Analysis of the solar flare and geomagnetic storm events in September 2017 and October 2021. Adv. Space Res..

[24] Luo X., Gu S., Lou Y., Xiong C., Chen B., Jin X. (2018). Assessing the performance of GPS precise point positioning under different geomagnetic storm conditions during solar cycle 24. Sensors.

[25] Wielgosz P., Kashani I., Grejner-Brzezinska D. (2005). Analysis of long-range network RTK during a severe ionospheric storm. J. Geod..

[26] Jacobsen K.S., Schäfer S. (2012). Observed effects of a geomagnetic storm on an RTK positioning network at high latitudes. J. Space Weather Space Clim..

[27] Jacobsen K.S., Andalsvik Y.L. (2016). Overview of the 2015 St. Patrick’s day storm and its consequences for RTK and PPP positioning in Norway. J. Space Weather Space Clim..

[28] Follestad A.F., Clausen L.B.N., Moen J.I., Jacobsen K.S. (2021). Latitudinal, Diurnal, and Seasonal Variations in the Accuracy of an RTK Positioning System and Its Relationship With Ionospheric Irregularities. Space Weather.

[29] Zhang J., Ren X., Mei D., Abdelaziz A., Zhang X., Pan G. (2024). Long-term analysis of NRTK positioning performances over one solar activity cycle from 2013 to 2023. GPS Solut..

[30] Li M., Zha J., Yuan Y., Zhao C. (2024). A unified model of multi-GNSS and multi-frequency precise point positioning for the joint estimation of ionospheric TEC and time-varying receiver code bias. J. Geod..

[31] Li W., Wang K., Yuan K. (2023). Performance and Consistency of Final Global Ionospheric Maps from Different IGS Analysis Centers. Remote Sens..

[32] Mannucci A., Wilson B., Yuan D., CHo H., Lindqwister U.J., Runge T.F. (1998). A global mapping technique for GPS-derived ionospheric total electron content measurements. Radio Sci..

[33] Zhou C., Yang L., Li B., Balz T. (2022). M_GIM: A MATLAB-based software for multi-system global and regional ionospheric modeling. GPS Solut..

[34] Agyei-Yeboah E., Fagundes P.R., Tardelli A., Pillat V.G., Vieira F., Bolzan M.J.A. (2025). Global ionospheric response to a G2 and a G3 geomagnetic storms of November 4 and 5 2023. Adv. Space Res..

[35] Luo X., Li Y., Dai X., Chen Z. (2025). Difference and threshold analysis of scintillation index S4c using GPS data from collocated stations at 30 s intervals during 2010–2023. GPS Solut..

[36] Takasu T. (2013). RTKLIB: An Open Source Program Package for GNSS Positioning.

[37] Saastamoinen J. (1972). Contributions to the theory of atmospheric refraction. Bull. Géodésique.

[38] Böhm J., Niell A., Tregoning P., Schuh H. (2006). Global Mapping Function (GMF): A new empirical mapping function based on numerical weather model data. Geophys. Res. Lett..

[39] Bojilova R., Mukhtarov P. (2023). Comparative analysis of global and regional ionospheric responses during two geomagnetic storms on 3 and 4 February 2022. Remote Sens..

